# Parity and Risk of Death from Gallbladder Cancer among a Cohort of Premenopausal Parous Women in Taiwan

**DOI:** 10.3390/ijerph120201864

**Published:** 2015-02-05

**Authors:** Te-Fu Chan, Chen-Hsuan Wu, Hui-Fen Chiu, Chun-Yuh Yang

**Affiliations:** 1 Graduate Institute of Medicine, College of Medicine, Kaohsiung Medical University, 100 Shih Chuan 1st RD, Kaohsiung 807, Taiwan; E-Mail: tefu.chan@msa.hinet.net; 2 Department of Obstetrics and Gynecology, Kaohsiung Medical University Hospital, 100 Shih‑Chuan 1st RD, Kaohsiung 807, Taiwan; 3 Department of Obstetrics and Gynecology, Kaohsiung Chang-Gung Memorial Hospital, No.123, DA-PI Rd. Niaosng Dist., Kaohsiung 833, Taiwan; E-Mail: chenhsuan5@gmail.com; 4 College of Medicine, Chang-Gung University, No.123, DA-PI Rd. Niaosng Dist., Kaohsiung 833, Taiwan; 5 Department of Pharmacology, College of Medicine, Kaohsiung Medical University, 100 Shih Chuan 1st RD, Kaohsiung 807, Taiwan; E-Mail: Chiu358@yahoo.com.tw; 6 Department of Public Health, College of Health Sciences, Kaohsiung Medical University, 100 Shih Chuan 1st RD, Kaohsiung 807, Taiwan; 7 Division of Environmental Health and Occupational Medicine, National Health Research Institute, 35 Keyan Road, Zhunan, Miaoli 350, Taiwan

**Keywords:** gallbladder cancer, parity, mortality, cohort study, Taiwan

## Abstract

Little epidemiologic research has been done on the etiology of gallbladder cancer (GC). This cohort study was undertaken to examine whether there is an association between parity and risk of death from GC. The study cohort consisted of 1,292,462 women who had a first and singleton childbirth between 1 January 1978 and 31 December 1987. We tracked each woman from the time of their first childbirth to 31 December 2009, and their vital status was ascertained by linking records with the computerized mortality database. Cox proportional hazard regression models were used to estimate the hazard ratios (HR) of death from GC associated with parity. There were 257 GC deaths during 34,980,246 person-years of follow-up. The mortality rate of GC was 0.73 cases per 100,000 person-years. As compared with women who had given birth to only one child, the adjusted HR was 1.20 (95% CI = 0.79–1.83) for women who had two children, 1.47 (95% CI = 0.95–2.29) for women who had three children, and 1.68 (95% CI = 0.99–2.85) for women with four or more births. There was a significant increasing trend in the adjusted HRs for GC with increasing parity. The findings suggested that premenopausal women of higher parity may increase the risk of death from GC.

## 1. Introduction

In Taiwan, gallbladder cancer (GC) is the 14th leading cause of cancer mortality for males and the 12th for females [1]. The age-adjusted mortality rate for GC was 1.9 per 100,000 among males and 1.5 among females in 2011. There is substantial geographic variation in GC mortality within the country.

GC is a relatively rare neoplasm and is considered to be a highly lethal disease [2]. There are no specific clinical symptoms or signs, and most patients have advanced stages at presentation. Prognosis for survival in 90% of cases is less than 5 years [3,4]. GC shows a marked geographic and ethnic variation [3,4,5]. These differences can have several interpretation, but they refer particularly to the worldwide distribution of gallstones, which are the most important risk factor for GC [4,5,6]. However, only 0.3%–3% of patients with cholelithiasis develop GC and approximately 20% of GC patients show no evidence of previous gallstones [3,6], and other risk factors have been proposed to play a role [4]. 

The incidence rates for GC and gallstones are two-fold higher in women [2,7], suggesting a possible role of female hormones on the development of GC [8]. Serum estrogen levels have been reported to rise about 100-fold during pregnancy [9]. Higher parity is therefore associated with increased lifetime exposure to estrogen. The role of parity in the etiology of GC in women have received only limited attention in the literature, and the results have been inconsistent. Some studies reported positive associations between increasing parity and the risk of GC [7,10,11,12,13] and others reported no association [14,15,16].

To date, there are limited data regarding the relationship between parity and GC [7,10,11,12,13,14,15,16]. We studied a cohort of women who experienced a first and singleton childbirth between 1 January 1978 and 31 December 1987 to explore further the association between parity and the risk of death from GC in Taiwan. 

## 2. Materials and Methods

### 2.1. Data Source

Registration of births is required by law in Taiwan. It is the responsibility of the parents or the family to register infant births at a local household registration office within 15 days. The Birth Registration System, which is managed by the Department of Interior, released computerized data on live births since 1978. The registration form, which requests information on maternal age, education, parity, gestational age, date of delivery, infant gender, and birth weight, is completed by the physician attending the delivery. Because most deliveries in Taiwan take place in either a hospital or clinic [17], the birth certificates are completed by physicians attending the delivery and it is mandatory to register all live births at local household registration offices, the birth registration data are considered complete, reliable and accurate. These data have been used in our previous studies [18,19]. In addition, this study was approved by the ethics review board of the Kaohsiung Medical University Hospital (KMUH-IRB-20130042).

### 2.2. Study Population

**Figure 1:**
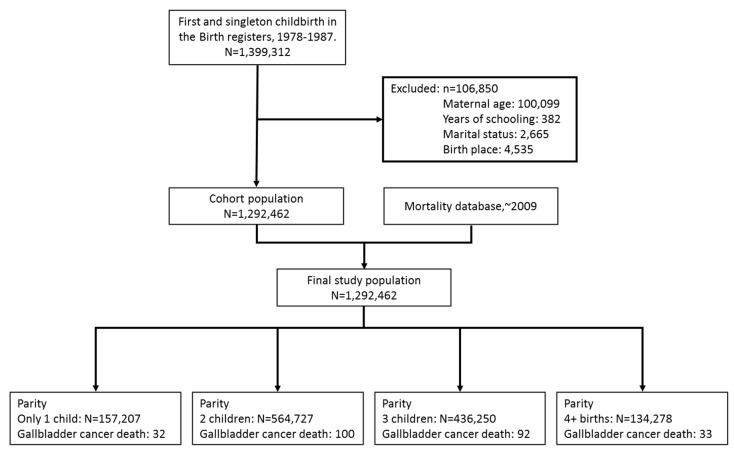
Flowchart of study subject selection.

The study cohort consisted of all women with a record of a first and singleton childbirth in the Birth Register between 1 January 1978 and 31 December 1987. Over this period, there were 1,399,312 first and singleton births in Taiwan. Information on any subsequent births was also retrieved from the Birth Register. Of the 1,399,312 primiparous women, 106,850 subjects were excluded because data were missing on at least one variable such as maternal age (*n =* 100,099), years of schooling (*n =* 382), marital status (*n =* 2665), or birth place (*n =* 4535). This left 1,292,462 women with complete information for the analysis (Figure 1). Their details have already been described in an earlier publication [20].

### 2.3. Follow-Up

Each woman has her own unique personal identification number. Using this number, we tracked each woman from the time of their first childbirth to 31 December 2009, and their vital status was ascertained by linking records with the computerized mortality database, identifying the date of any deaths occurring in this cohort. Of the 1,292,462 women followed, none had a missing personal identification number; therefore, all cohort members were followed up. Since it is mandatory to register death certificates at local household registration offices, the death statistics in Taiwan were considered to be highly accurate and complete [20,21].

### 2.4. Statistics

We categorized parity (the number of children recorded in the last childbirth record of each woman registered during follow-up) into 4 categories: 1, 2, 3, 4 or more. We compared selected baseline characteristics of the cohort with regard to parity using chi-square tests and analysis of variance, as appropriate. The person-years of follow-up for each woman was calculated from the date of first childbirth to the date of death or 31 December 2009. Death rates were calculated by dividing the number of deaths from GC by the number of person-years of follow-up. We used the Cox proportional hazard regression models with time-dependent covariates to estimate the hazard ratios (HRs) of death from GC associated with parity. The 95% confidence intervals (CIs) for the HRs were also calculated. GC is defined according to the International Classification of Disease, Injury, and Causes of Death (9th revision) (ICD code 156.0). We used 2 Cox proportional hazards models: an age-adjusted model and a multivariate-adjusted model that was additionally adjusted for marital status (married, unmarried), years of schooling (≤9, >9 years), and birth place (hospital/clinic, home/other). The proportional hazards assumption was assessed for all abovementioned variables, and no violations were observed. To test for trends in risk with increasing levels of the exposure of interest (parity), we assigned the categorical variables their ordinal number for parity and then fitted the assigned values as a continuous variable in the risk models. We then evaluated the statistical significance of the corresponding coefficient using the Wald test [22]. Analyses were performed using the SAS statistical package (version 9.2, SAS Institute Inc., Cary, NC, USA). All statistical tests were two-sided; *p* values of less than <0.05 were considered to be statistically significant.

## 3. Results

Altogether 1,292,462 primiparous women with complete information were included in the analysis. A total of 34,980,246 person-years were observed during the follow-up period from the time of their first childbirth to 31 December 2009. There were 257 GC deaths, yielding a mortality rate of 0.73 cases per 100,000 person-years. 

Table 1 shows the baseline characteristics of the study population by parity. Compared with women who had given birth to only one child, women with four or more children were more likely to have a lower educational level, to be younger at first birth, and to have a lower rate of being born in a hospital or clinic.

Table 2 presents the hazard ratios (HRs) of GC mortality by parity. After adjustment for age at first birth, the HRs for GC death was 1.12 (95% CI = 0.75–1.69) for women who had 2 children, 1.53 (95% CI = 0.99–2.35) for women who had 3 children, and 1.90 (95% CI = 1.13–3.20) for women with 4 or more births, as compared with who had given birth to 1 child. A trend of increasing risk of death from GC was seen with increasing parity (*p* for trend = 0.0023). In the multivariate-adjusted model (*i.e.*, adjusted for age at first birth, marital status, years of schooling, and birth place), the adjusted HR was 1.20 (95% CI = 0.79–1.83) for women who had 2 children, 1.47 (95% CI = 0.95–2.29) for women who had 3 children, and 1.68 (95% CI = 0.99–2.85) for women with 4 or more births, when compared with women who had given birth to only 1 child. There was a statistically significant increasing trend in the adjusted HRs for GC mortality with increasing parity (*p =* 0.025).

**Table 1:** Demographic characteristics of the study cohort.

Parameters	Parity								*p*-Value
	1 (*n =* 157,207)		2 (*n =* 564,727)		3 (*n =* 436,250)		4+ (*n =* 134,278)		
Age at recruitment (1st birth)	26.38 ± 4.43		24.86 ± 3.30		23.49 ± 2.95		22.44 ± 2.95		<0.0001
Marital status									
Married	146,022	(92.89)	554,810	(98.24)	429,239	(98.39)	130,544	(97.22)	<0.0001
Not married	11,185	(7.11)	9917	(1.76)	7011	(1.61)	3734	(2.78)	
Years of schooling									
≤9 year	72,090	(45.86)	258,361	(45.75)	285,737	(65.50)	106,330	(79.19)	<0.0001
>9 year	85,117	(54.14)	306,366	(54.25)	150,513	(34.50)	27,948	(20.81)	
Birth place									
Hospital/clinic	153,167	(97.43)	553,930	(98.09)	416,492	(95.47)	122,336	(91.11)	<0.0001
Home/other	4040	(2.57)	10,797	(1.91)	19,758	(4.53)	11,942	(8.89)	

**Table 2:** Association between parity and hazard ratio of death from gallbladder cancer over a 32-year follow-up period.

Parity	No. of Subjects	No. of Death from Gallbladder Cancer	Follow-Up Person-Years	Mortality Rate (Per 100,000 Person-Years)	Age-Adjusted Hazard Ratio (95% CI)	Multivariate-Adjusted Hazard Ratio * (95% CI)
1	157207	32	4170772.33	0.77	1.00	1.00
2	564727	100	15124112.33	0.66	1.12 (0.75~1.69)	1.20 (0.79~1.83)
3	436250	92	11925297.25	0.77	1.53 (0.99~2.35)	1.47 (0.95~2.29)
4+	134278	33	3760064.08	0.88	1.90 (1.13~3.20)	1.68 (0.99~2.85)
					*p =* 0.0023 for linear trend	*p =* 0.0246 for linear trend

* Adjusted for age, marital status, years of schooling, and birth place.

## 4. Discussion and Conclusions

Probably due to its infrequent occurrence and rapid fatal outcome, little epidemiologic research has been done on the etiology of GC. To our knowledge, this is the first prospective study to examine the relationship between parity and the risk of death from GC. Previous studies all used case-control designs [7,10,11,12,13,14,15,16].

In this prospective cohort study, we found that women with higher parity have an increased risk of death from GC. Our findings of an increase in GC mortality risk associated with higher parity is in agreement with the results of some studies [7,10,11,12,13]; however, others studies have reported no association with higher parity [14,15,16].

The mechanisms by which increased parity may increase the risk of future development of GC in women remains unclear. Pregnancy elevates serum estrogen levels approximately 100-fold [9]. Increasing parity is associated with an overall increase in lifetime exposure to sex hormones. The hormonal changes may both alter the composition of bile and impair biliary motility to promote the development of gallstones [23], which is also associated with increasing parity and is the most important risk factor for GC [4,5,6,24], although a direct effect of estrogens or secondary bile acids on the biliary epithelium is possible. In addition, estrogens increase biliary cholesterol saturation [25,26] and ultrasound studies show increase in biliary sludge and stones in pregnant women [27]. Thus our findings could be explained by the fact that with each additional birth, there is a repeated exposure to high estrogen and progesterone levels, resulting in a cumulative increase in the likelihood of development of gallstones or the enlargement of pre-existing gallstones and therefore an increased potential for GC later in life [26]. Furthermore, sex hormones have been demonstrated to influence the functioning of normal gallbladder. Progesterone may influence sphincter of Oddi function which, in turn, affects gallbladder emptying [28]. The mechanism by which cholelithiasis contributes to GC is not clear. Gallstones cause mechanical irritation and provides a nidus for infection in the gallbladder. Chronic infection incites chronic inflammation that may result in the metaplasia-dysplasia sequence ending up in carcinogenesis [29], a hypothesis that is supported by the strong long-term protective effective of removal of the gallbladder on the development of bile duct cancer [30].

Mortality data have been widely used to generate epidemiologic hypotheses, despite their inherent limitations. The completeness and accuracy of the death registration system should be evaluated before any conclusion based on the mortality analysis is made. In the event of a death in Taiwan, the decedent’s family is required to obtain a death certificate from the hospital or local community clinic, which then must be submitted to the household registration office in order to cancel the decedent's household registration. The death certificate is required in order to have the decedent's body buried or cremated. Death certificates must be completed by physicians in Taiwan. It is also mandatory to register all deaths at local household registration offices, the death registration is accurate, reliable and complete. The complete population coverage and follow-up made possible by the national identification number has left the study without selection bias. The possibility of bias in the selection of data on parity is also unlikely to be a concern.

Information on emigration was not available in this study. The results are based on observation of the women who did not emigrate. Our study populations is very stable in emigration compared with populations in industrialized countries, and the follow-up is very complete mainly because the use of the national identification number, which also minimized the possibility of selection bias in this study. Furthermore, it seems unlikely that emigration would be correlated with parity. We therefore believe that emigration should not have a substantial effect on the association we observed.

Taiwan is a small island with a convenient communication network. It is believed that all GC cases had access to medical care. Mortality data rather than data on inpatient cases was used to assess the association between parity and GC mortality in this study. The mortality of a disease is a function of its incidence and fatality. Survival is less than 5 years in 90% of cases [3,4]. Deaths from GC may therefore be regarded as a reasonable proxy for GC incidence.

Our data takes into account the effect of the number of children on the risk of mortality from GC. The birth registration system in Taiwan covers only live births and does not include abortions and stillbirths. Therefore we were unable to examine the possible role of gravidity on the risk of death from GC. Our study design only allowed for the study of mortality among parous women. Again, we were unable to examine the possible role of nulliparity on the risk of death from GC because the birth registry ascertained births rather than pregnancies. The generalizability of our findings is thus limited. Further study in independent cohorts with the inclusion of non-parous women for follow-up studies of the association between parity and the risk of death from GC are needed.

Women aged 65 and older had the highest mortality rates of GC in Taiwan. The mean age at death for GC was 46.92 ± 6.03 years in this study. Women included in this study were still young and had yet not reached the age associated with the highest GC risk, therefore the generalizability of our findings may be limited.

Reports of oral contraceptives (OCs) use in relation to GC do not support a statistically significant association [7,12,15,31,32,33]. The limited studies conducted have not found a consistent association of hormone replacement therapy (HRT) with GC risk [15,34,35]. We were unable to adjust for these two hormonal factors in the current study due to the lack of available data. Since the use of OC and HRT are low in Taiwan compared with Western countries [36,37], the confounding effect resulting from these two factors should be small, if any exists at all. Furthermore, the limited exposure to OCs and HRT allowed us to assess parity independently of exogenous hormones.

Obesity is a major risk factor for GC [4,5,15,32,38,39], probably through the strong relationship between overweight and the occurrence of gallstones [40]. There is no information available on obesity for individual study subjects and thus it could not be adjusted for in the analysis. However, obesity is common in women who have had several pregnancies [41,42,43]. Hence, the actual risk reduction of GC mortality in women with higher parity would have been larger than that shown if we had been able to adjust for obesity. Nonetheless, the lack of information on obesity should be regarded as a limitation of this study because obesity is a major risk factor for GC.

History of gallstones was the strongest risk factor for GC. On a global level, a strong geographic correlation exists between gallstone prevalence and GC incidence [4,5,6]. In a review and meta-analysis of risk factors for GC, a pooled relative risk of 4.9 was estimated for history of gallstones [4]. We were unable to adjust for this factor due to the lack of available data. The lack of information on history of gallstones should be regarded as a limitation of this study. The frequency of cholecystectomy operations would affect the incidence of GC and likely explains some of the observed variations among countries and over time [4,5]. Cholecystectomy is also associated with a reduced risk of GC [4,30,44]. Again, we were unable to adjust for this factor due to the lack of available data. Access to cholecystectomy accounts for at least part of socioeconomic differentials. We did not have information on access to cholecystectomy of our study subjects, and we could not directly adjust for it in the analysis. However, years of schooling was used as a proxy for socioeconomic status and was included as a control variable in the multivariate analysis. We therefore may have partially indirectly adjusted for the confounding effect of access to cholecystectomy. 

In conclusion, we found that there was a trend for increasing parity to be associated with increasing mortality risk for GC among a cohort of young parous women. Our study is the first cohort study to show a positive association between parity and risk of death from GC. Nonetheless, because the literatures to date lacks consistent evidence of an association between parity and GC mortality risks. More work will be needed to clarify the effect of parity on GC mortality risk.
